# Anxiety and Depression in COVID Survivors - A Cross-Sectional Study Six Months Past the Acute Illness

**DOI:** 10.7759/cureus.42971

**Published:** 2023-08-04

**Authors:** Vijaya Chandra R Avula, Sridhar Amalakanti, Shashidhara M, Afreen Fasiha

**Affiliations:** 1 Psychiatry, All India Institute of Medical Sciences, Mangalagiri, IND; 2 General Medicine, All India Institute of Medical Sciences, Mangalagiri, IND; 3 Pulmonology, All India Institute of Medical Sciences, Mangalagiri, IND

**Keywords:** long haul covid-19, post-acute covid-19 syndromes, long covid, post-covid condition, post covid anxiety, post covid depression, covid-19

## Abstract

Background

Studies exploring the long-term psychiatric sequelae of COVID-19 are sparse. We aimed to assess depression and anxiety six months after recovery and the association between disease severity and psychiatric sequelae.

Material and methods

Our study was a comparative study conducted on COVID-19 disease survivors at a tertiary hospital. We compared Hamilton Depression Rating Scale (HDRS) scores and Hamilton Anxiety Rating Scale (HAM-A) scores between mild (n=50), moderate (n=50), severe cases (n=50), and controls (n=50). We assessed the severity of depression and anxiety using the HDRS and the HAM-A. Cases: First-time COVID-19 survivors six months post-recovery. Cases were healthy pre-COVID. To the study date, controls were negative for COVID-19 reverse transcription polymerase chain reaction (RT-PCR).

Results

Our study of 200 subjects indicated that mean (mean, SD) HAM-A scores in cases (14.7, 5.6) were higher than controls (7.9, 1.7), and HDRS scores in cases (17.3, 6.3) were higher than controls (7.5, 1.9). HAM-A scores in severe cases (19.5, 4.8) were higher than in moderate cases (17.0, 3.9), and scores in moderate cases were higher than in mild cases (10.6, 2.4). HDRS scores in severe cases (22.5, 5.9) were higher than in moderate cases (16.4, 3.2), and scores in moderate cases were higher than in mild cases (14.4, 2.5). Among the cases, there was a positive correlation between the Hamilton Anxiety Rating Scale (HAM-A) and Hamilton Depression Rating Scale (HDRS) scores and the duration of hospital stay, ICU stay, and use of invasive oxygen. These parameters were not applicable to the controls; hence, they were not included in the comparative analysis.

Conclusions

Patients, even after six months of recovery from COVID-19, had symptoms of anxiety and depression. The severity of anxiety and depression in survivors correlates with the severity of the COVID-19 disease.

## Introduction

India reported its first case of COVID-19 in January 2020 [1]. According to 'Our World in Data', the first wave of the virus hit India in March 2020, peaking in September 2020 with over 90,000 daily cases. By February 2021, daily cases had dropped to 10,000. The second wave began in April 2021 and peaked in May 2021, with 2.96 million new cases. The number of positive cases returned to normal levels in September 2021 [2].

COVID-19, caused by the SARS-CoV-2 virus, typically presents with mild or no symptoms [3]. However, 20% of patients may develop severe complications like acute respiratory distress syndrome and hypercoagulability due to a virus-triggered cytokine storm. While the direct impact of SARS-CoV-2 on the brain and resulting neurological and psychiatric symptoms remains uncertain, the virus-induced cytokine storm, characterized by increased levels of serum C-reactive protein, interleukins (IL-1, IL-6, IL-10), and tumor necrosis factor (TNF-alpha), is associated with psychiatric symptoms, including depression, anxiety, stress, and suicidality [4].

Quarantine, job loss, social dynamics change, health uncertainty, media rumors, limited psychiatric help, and migration contributed to psychosocial challenges during the COVID-19 pandemic [5].

Liu et al.'s meta-analysis found high prevalence rates of anxiety (32%), depression (27%), insomnia (30%), and post-traumatic stress disorder (PTSD) (17%) in individuals during the COVID-19 pandemic. Confirmed and suspected COVID-19 cases had the highest rates of anxiety (64%) and depression (55%). [6] Kaur et al.'s study during the second wave found that a significant proportion of participants reported symptoms resembling PTSD (44%), depression (48.87%), anxiety (65.56%), stress (22.09%), and disturbed sleep (11.27%) [5].

Survivors of SARS and Middle East respiratory syndrome (MERS) have experienced lasting neuropsychiatric issues, including a prevalence of post-traumatic disorder in 32.2% of cases, depression in 14.9%, and anxiety in 14.8% [7,8]. Even during the COVID-19 pandemic, patients had persistent lung dysfunction and psychological and cognitive symptoms. Among survivors of COVID-19, the median frequency of fatigue or musculoskeletal weakness was 63%, anxiety or depression was 23%, and insomnia was reported by 29%. The National Health Services (NHS) in England has identified various symptoms associated with Long COVID syndrome, which includes psychiatric symptoms such as insomnia, depression, and anxiety [9].

Cytokine storm-induced hyperinflammation and hyper-coagulability in COVID-19 patients leading to brain damage is closely linked to neuropsychiatric symptoms [10]. Given the scarcity of studies on psychological symptoms in COVID-19 recovery patients in India, our objective was to compare the anxiety and depression scores among individuals who had experienced mild, moderate, and severe COVID-19 disease.

## Materials and methods

Our study was conducted at the COVID-19 recovery clinic following institutional ethical clearance at All India Institute Of Medical Science (AIIMS), Mangalagiri - AIIMS/MG/IEC/2021-22/127. The study occurred from November 2021 to January 2022, and participants provided written informed consent. Controls were individuals who had never tested positive for COVID-19 through reverse transcription polymerase chain reaction (RT-PCR) until the day of the interview. Cases included individuals who had tested positive for COVID-19 through RT-PCR at least 180 days before the interview. We categorized the cases into mild, moderate, and severe COVID-19 based on the severity classification and management guidelines provided by the Indian Council of Medical Research (ICMR).

Exclusion criteria for both cases and controls included a previous history of chronic physical illnesses such as stroke, chronic obstructive pulmonary disease (COPD), diabetes mellitus, chronic kidney disease (CKD), and any other chronic conditions requiring long-term medications before the illness. Individuals with a previous or current history of psychiatric illness were also excluded from the study.

Patients without proper case records to accurately classify them into mild, moderate, and severe COVID-19 disease categories were excluded. Furthermore, individuals with a prior diagnosis of COVID-19 before the second wave (i.e., May 2021 to September 2021) were also excluded from the study.

We retrieved demographic information, including age, sex, pre-existing comorbidities, date of diagnosis, date of discharge, duration of hospital stay, duration of ICU stay, type of oxygen support (invasive, non-invasive), and details of medication use (steroids and remdesivir) from the patient discharge records. For individuals in home isolation or quarantine at government centers, we collected data on the duration of home isolation or quarantine and the medications administered during that period.

We assessed levels of depression and anxiety using two standardized scales: the Hamilton Anxiety Rating Scale (HAM-A) and the Hamilton Depression Rating Scale (HDRS). The HAM-A questionnaire consists of 14 items, and scores were categorized as follows: 8-14 indicated mild anxiety, 15-24 indicated moderate anxiety, scores greater than 24 predicted severe anxiety, and scores below 7 indicated the absence of anxiety [11]. The Hamilton Depression Rating Scale (HDRS) consists of 17 items, and scores on this scale are categorized as follows: a score below 7 indicates no depression, scores between 8 and 16 indicate mild depression, scores between 17 and 23 indicate moderate depression and scores above 24 indicate severe depression [12].

Sampling and sample size

We enrolled consenting cases and controls using a convenient sample. To detect a minimum two-point difference in HAM-A/HDRS scores between post-COVID subjects and controls, we determined a sample size of 36 per group based on the mean scores in previous publications with a power of 80% at 95% CI [13]. We included 50 controls and 150 cases, with 50 cases each in the mild, moderate, and severe COVID-19 history groups.

Statistical analysis

Data analysis was performed using Statistical Package for the Social Sciences (SPSS) version 22.0 (IBM Inc, Armonk, New York). Parametric tests were utilized to compare means, as the age distribution of controls and cases followed a normal distribution. Descriptive statistics were employed, including proportions for qualitative factors and mean and standard deviations for quantitative factors. The chi-square test and analysis of variance (ANOVA) were used to compare the HAM-A and HDRS scores for qualitative and quantitative variables, respectively. Pearson correlations were used to measure the association between variables. A significance level of p<0.05 was considered statistically significant for all tests.

## Results

A total of 200 subjects were analyzed. A Chi-squared test for the homogeneity to determine males and females in four groups were equal in proportion, and the proportion of males and females did not differ significantly χ2(3, 200) =5, p=0.2. The mean ages of males and females in the four groups did not differ significantly F (1,197) =0.7, p=0.4 on ANOVA. The combined mean age of male and female participants in the four groups did not differ significantly F (3,196) =1.9, p=0.1 (Table 1).

**Table 1:** Mean age and proportion of males and females in the study across various severity groups a - proportion of female and male participants were comparable χ2 =5 df=3 p=0.2; b - mean age of females and males in all categories were comparable‑ F=0.7 df=1 p=0.4; c - mean age of the control, mild, moderate, and severe groups were comparable F=1.9 df=3 p=0.1

Severity of disease	Proportions of males and females in each category		Total sample size	Mean age of Males and Females in each category		Mean age of participants, mean (SD)
	Males N (%)	Females N (%)		Mean age males, mean (SD)	Mean age of females, mean (SD)	
Controls	21 (42%)	29 (58%)_ a_	50	42 (13)	49 (12)_b_	46 (12)_c_
Mild	26 (52%)	24 (48%)_ a_	50	44 (12)	39 (9)_b_	41 (11)_c_
Moderate	28 (56%)	22 (44%)_ a_	50	43 (7)	48 (7)_b_	45 (9)c
Severe	32 (64%)	18 (36%)_ a_	50	45 (9)	43 (9)_b_	44 (10)_c_

**Figure 1:**
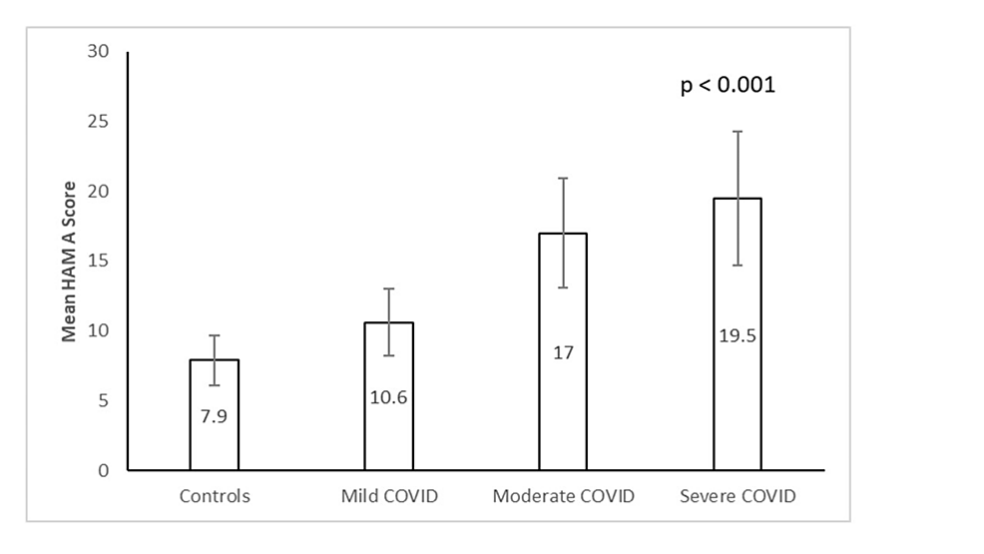
HAM-A scores across the groups HAM-A - Hamilton Anxiety Rating Scale

**Figure 2:**
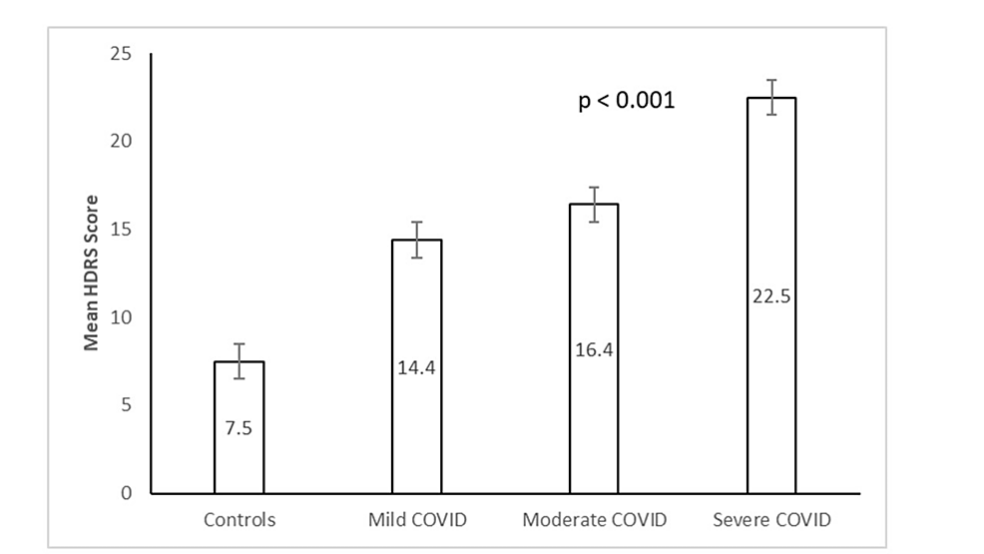
HDRS scores across the groups HDRS - Hamilton Depression Rating Scale

The mean scores of HAM-A and HDRS across the four groups differed significantly using ANOVA (HAM-A: F (3, 196) = 124.1, η2 = 0.7, p<0.001; HDRS: F (3, 196) = 140.22, η2 = 0.7, p<0.001). Levene's test for homogeneity of variances was significant (p<0.001), indicating that the groups were not homogeneous. The Welch test revealed significant differences in the mean HAM-A scores among the four groups (p<0.001). Additionally, significant differences were observed in the mean HDRS scores among the four groups (p<0.001). Post hoc comparison using the Games-Howell test revealed that the severe group had significantly higher mean scores on the HAM-A and HDRS than the moderate group (p<0.02). The moderate group exhibited higher scores than the mild group (p<0.001), and the mild group had higher scores than the controls (p<0.001). These findings indicate a positive correlation between the severity of the disease and the scores on HAM-A and HDRS (Figures 1, 2).

Among the participants, females had a higher mean HAM-A score (15.7 ± 7.2) compared to males (13.2 ± 5.2), and this difference was statistically significant (p=0.01). However, there was no significant difference in the mean HDRS score between females (16.6 ± 6.6) and males (17.4 ± 6.8; p=0.5).

We assessed the impact of different types of oxygen support on HAM-A and HDRS scores in COVID-19 cases. Four groups were analyzed: "no oxygen support" (n=48), "nasal oxygen" (n=39), "bilevel positive airway pressure" (n=27), and "ventilator support" (n=36). Significant differences were found in mean HAM-A scores (F (2,146) = 20.2; p<0.001) and mean HDRS scores (F (2,146) = 61.4; p<0.001) between the groups. Post hoc analysis using the Games-Howell test revealed that patients receiving ventilator support had significantly higher mean HAM-A scores (p<0.001). The bilevel positive airway pressure (BiPAP) group also had higher HAM-A scores compared to the no-oxygen support group (p = 0.02). Mean HDRS scores were higher in the ventilator support group (p<0.001) compared to the BiPAP group, while the BiPAP group had higher scores than the nasal oxygen group (p<0.001). The group receiving nasal oxygen had higher scores than the no-oxygen support group (p<0.001). The HAM-A scores between the no oxygen and nasal oxygen groups were comparable (p=0.5), as were the scores between the nasal oxygen and BiPAP groups (p=0.3). These findings suggest that the invasiveness of the oxygen support was related to higher HDRS scores, and both the BiPAP and ventilator-supported groups had higher HAM-A scores (Table 2).

**Table 2:** Comparison of HAM-A and HDRS scores in disease population grouped by O2 treatment HAM-A - Hamilton Anxiety Rating Scale; HDRS - Hamilton Depression Rating Scalev BiPAP - bilevel positive airway pressure

		N	Mean	SD	95% CI Mean		F-value
					LL	UL	
HAM-A	No O_2 _support	48	13	4.3	11.8	14.3	F (3,146) =20.2; h^2=^0.3; p<0.001
	Nasal oxygen	39	14.2	4	12.9	15.5	
	BiPAP	27	16.2	4.6	14.4	18	
	Ventilator	36	20.5	5.3	18.7	22.3	
HDRS	No O_2 _support	48	13.5	2.2	12.9	14.2	F (3,146)=61.4; h^2^_=_0.6; p<0.001
	Nasal oxygen	39	16.2	3.1	15.2	17.2	
	BiPAP	27	19.4	2.2	18.5	20.2	
	Ventilator	36	24	5.8	22	25.9	

Regarding drug treatment, the study population was divided into four groups: no remdesivir or steroids (n=42), remdesivir only (n=54), remdesivir and steroids (n=24), and steroids only (n=30). ANOVA analysis showed significant differences in mean HAM-A scores (F (3,196) = 30.8, p<0.001) and mean HDRS scores (F (3,196) = 33.0, p<0.001) among these groups. Post hoc analysis revealed that the group without any drug treatment had significantly lower HAM-A and HDRS scores compared to the other groups (p<0.001). However, there were no significant differences in HAM-A and HDRS scores among the groups receiving different drug treatments.

Pearson correlations showed that HDRS scores positively correlated with the duration of hospital stay (r=0.46, p<0.001) and ICU stay (r=0.46, p<0.001). Similarly, HAM-A scores positively correlated with the duration of hospital stay (r=0.54, p<0.001) and ICU stay (r=0.47, p<0.001). Factors such as age, SpO2 levels, and drug use did not significantly correlate with higher HAM-A and HDRS scores. However, a longer length of stay in the hospital and ICU was associated with higher scores on these scales.

## Discussion

Our study explored the relationship between disease severity and post-COVID anxiety and depression. Results revealed higher levels of anxiety and depression in recovered COVID-19 patients compared to the control group, consistent with previous research. Specifically, patients with severe COVID-19 admitted to the ICU exhibited more severe anxiety and depressive symptoms than those in the observation ward with moderate disease. Patients with mild disease showed the lowest scores on the HDRS and HAMA. 

In a retrospective cohort study of 236,379 COVID-19 survivors, anxiety disorders were observed in 17.5% of non-hospitalized individuals, 16.9% in the hospitalized group, and 19.2% in those admitted to the ICU. Mood disorders were reported in 13.1%, 14.1%, and 15.9% of the respective groups. These findings highlight the increased risk of anxiety and mood disorders associated with illness severity and the need for hospitalization or ICU admission [14].

Considering the concept of post-intensive care syndrome (PICS) [15], our study suggests that the observed depressive and anxiety symptoms in patients who survived severe COVID-19 may be a manifestation of PICS.

Our study findings support previous research, suggesting that females who have recovered from COVID-19 may be more susceptible to experiencing anxiety compared to males. The LONG-COVID-EXP-CM Multicenter Study [16] specifically identified female sex as a risk factor for developing anxiety, corroborating our observations. Similarly, a UK-based study focusing on discharged COVID-19 patients [17] found higher levels of anxiety and depression among females, aligning with our results and indicating persistent mental health challenges post-hospital discharge.

A one-year follow-up study of COVID-19-recovered patients [18] further supported our findings by identifying the female sex as a risk factor for post-COVID depression and anxiety. Additionally, a referenced study [19] highlighted the increased susceptibility of females to post-recovery depression. These studies collectively emphasize the vulnerability of females to mental health issues following COVID-19.

It is noteworthy that depression and anxiety are recognized symptoms in the National Institute for Health and Care Excellence (NICE) guidelines for long COVID-19. Sexual dimorphism in response to viral infections may be influenced by X chromosome-linked genes, impacting susceptibility to viral infections and autoimmune diseases [20]. This could explain the differential mental health outcomes between males and females observed in our study.

In a longitudinal cohort study by Huang et al. [21], which examined the one-year outcome in hospital survivors, anxiety or depression was measured using the European Quality of Life 5 Dimensions 5 Level Version (EQ-5D-5L) scale at six months and one year after hospitalization. The study found that among admitted patients who did not require any oxygen support, the prevalence of anxiety or depression was 24% at six months and 25% at one year. Patients needing non-invasive supplemental oxygen had a prevalence of 21% at six months and 26% at one year. In contrast, patients requiring invasive supplemental oxygen had a higher prevalence of anxiety or depression, with rates of 36% at six months and 29% at one year after recovery [21].

An Italian study [22] found that patients receiving ventilatory support of oxygen reported significantly higher levels of anxiety and depression. Consistent with these findings, our study revealed that patients who received ventilator oxygen support exhibited more severe anxiety and depression compared to those receiving BiPAP, nasal oxygen, or spontaneous breathing.

A study conducted on African-Americans [23] reported that four out of ten patients admitted to the ICU and on mechanical ventilation developed depression within ninety days of discharge, with a longer ICU stay being a significant risk factor. In our study, we also observed that patients on mechanical ventilation scored higher on depressive scores compared to those on less invasive methods such as BiPAP, nasal oxygen, or no oxygen support.

Our study findings indicate that there was no significant difference in anxiety and depression levels between patients treated with remdesivir alone and those treated with a combination of remdesivir and corticosteroids. This aligns with the study by Huang et al. [21], which found that corticosteroid use during COVID-19 admission did not impact the development of anxiety or depression at six months and one year after recovery. Similarly, a study on risk factors in COVID-19 patients discharged from the hospital did not find any association between the use of corticosteroids and the severity of anxiety and depression [14]. Another study conducted in South Korea on factors associated with depression and anxiety after discharge also did not show any association with the use of remdesivir [24].

Furthermore, our study revealed a positive correlation between the duration of hospital stay and scores on the HAMA and HDRS. This finding is consistent with a cross-sectional study evaluating depression and anxiety one month after recovery [25]. Additionally, ICU stay was positively correlated with depression and anxiety levels six months after discharge, which is in line with a study conducted on COVID-19-recovered patients [26].

Limitations of the study include the specific timeframe (November 2021 to January 2022), which may restrict the applicability of the findings to other time periods or changes in COVID-19 management. Relying on self-reporting and patient recall may introduce recall bias and inaccuracies in the collected information. The study also does not compare the development of anxiety and depression after any major illness leading to hospitalization, including post-ICU discharge. The sample size may have been small, which could limit the representativeness and statistical power of the study, particularly true of the individual subgroup analyses. Unaccounted inherent differences between cases and controls could have influenced the results. Excluding patients with previous or current psychiatric illnesses may limit the generalizability of the findings to a broader population of COVID-19 survivors. Finally, the study lacked long-term follow-up, potentially missing important long-term effects or outcomes beyond the 180-day mark. It is important for future research to address these limitations to improve the validity and generalizability of the findings.

## Conclusions

In conclusion, our study supports previous research that indicates a higher prevalence of anxiety and depression among patients who have recovered from COVID-19, particularly in those with severe disease and those who require ICU or ventilator support. Females may be more susceptible to experiencing anxiety and depression post-recovery, and longer hospital stays and ICU admissions are associated with higher levels of anxiety and depression. However, our study did not find a significant difference in anxiety and depression levels between patients treated with remdesivir alone or combined with corticosteroids. These findings highlight the need for targeted mental health interventions for COVID-19 survivors, particularly those with severe disease and females. Understanding the risk factors and impact on mental health outcomes can aid in developing effective strategies for long-term care and support.
